# Nursing students’ perception on their readiness to combat gender-based violence during the COVID-19 pandemic

**DOI:** 10.4102/hsag.v27i0.1968

**Published:** 2022-11-07

**Authors:** Nestor Tomas, Gideon Rupare

**Affiliations:** 1 Department of General Nursing Science, Faculty of Health and Veterinary Medicine, University of Namibia, Rundu, Namibia

**Keywords:** readiness, nursing student, gender-based violence, domestic violence, perception, COVID-19, Namibia

## Abstract

**Background:**

Gender-based violence (GBV) is a critical public health concern, demanding for global coordinated efforts. While nursing students form part of a healthcare team with significant roles in identifying and responding to abused victims, studies assessing their readiness to combat GBV are limited in Namibia.

**Aim:**

To assess undergraduate nursing students’ perceptions and to determine the relationship of the study variables to readiness to combat GBV during the coronavirus disease 2019 (COVID-19) pandemic in Namibia.

**Setting:**

This study was conducted at a university satellite campus in Namibia.

**Methods:**

A cross-sectional survey was used to collect data from 105 third and fourth-year undergraduate nursing students using ADKAR model. Data were analysed using Statistical Package for the Social Sciences, version 27.0, for descriptive statistics and logistic regression in determining relationships between study variables.

**Results:**

The study results show a mean readiness of 1.65 ± 0.19. Most respondents perceived themselves ready (73.3%), whereas 26.7% were not. Readiness was common with reinforcement (89.5%) and awareness (84.8%), knowledge (81.9%) and desire (76.2%) subscales. Predictors of readiness in this study were gender and age (*R*^2^ = 0.40; *R*^2^ = 0.37; *p* ≤ 0.05).

**Conclusion:**

The results of this study highlight that age and gender were significant predictors for readiness among the undergraduate students in Namibia. The results highlight the importance of GBV education in a nursing curriculum. A qualitative design is recommended for future studies.

**Contribution:**

The results of this study will support global efforts in strengthening the health system response on GBV incidences.

## Introduction

Gender-based violence (GBV) remains a critical public health challenge, which led to United Nations (UN) calling for member countries to prioritise emergency response systems (Mittal & Singh 2020:4). Gender-based violence is defined as an act of sexual, physical, emotional and mental harm towards any person regardless of gender or sex (Dlamini 2021:1; Muluneh et al. 2020:1). Muluneh et al. (2020:1) stressed that GBV takes various forms, and its definition is based on the perpetrator and victim relationship, that is, intimate partner violence (IPV) and non-intimate partner violence (NIPV). Current evidence suggest that GBV remains one of the threat to public health and affect all social class and cultural groups, women and children in particular (Dlamini 2021:2; John et al. 2020:1; Montesanti 2015:1). Although nursing students are members of multidisciplinary health teams with a significant role to identify and respond to GBV victims, studies assessing their readiness to combat GBV are limited in Namibia.

## Background

Globally, one in three women experienced physical violence in their lifetime (Noble et al. 2019:1; UNFPA 2019:1). This translates to about 243 million cases being reported globally, including 40% child marriage (Buttell & Ferreira 2020:1; UN Women 2020:1). Recent studies highlight a surge in GBV cases in developed countries with an increase of 20% – 50% between 2019 and mid-March 2020 (Ertan et al. 2020:3; Kagi 2020:1; Lundin et al. 2020:1; Mittal & Singh 2020:3), with sexism and harassment against women being the common form of GBV in Europe (Union 2018). Despite tangible improvements made in implementing GBV policies in industrial countries, there is a need for systematic identification of conditions in which policies on GBV can significantly contribute to dismantling sexual hierarchical power, particularly in nursing education (Engeli & Mazur 2018; Paganetti 2022).

The prevalence of GBV in sub-Saharan Africa ranges from 42.3% to 67.7%, with 44% being the IPV cases among women, where 25.9% are physical abuse, 29.4% being emotional abuse while 18.8% are sexual violence (Beyene et al. 2019; Muluneh et al. 2020:1). Statistics in Namibia show that about 5961 cases of GBV have been recorded during the 2019/2020 period (Amakali 2021:1). Media reported the surge in GBV cases as ‘shadow pandemic: GBV’ (The Namibian article:1), with 120 cases of GBV involving girls were reported in 2019 alone (Mukaiwa 2021:5).

Several studies cited various factors fuelling GBV (Ertan et al. 2020:3; Kagi 2020:1; Mittal & Singh 2020:3). Pandemics were found to fuel GBV because of its influence on socio-economic and psychological status (Mak et al. 2009:1). Chime, Nduagubam and Orji (2022:2) state that residing in a conflict or post conflict country, persistence of harmful cultural practices, alcohol abuse, higher level of poverty, social and economic exclusions and religion could fuel violence against the vulnerable groups. Similarly, IPV was particularly common when a perpetrator is a source of livelihood for the victim (Briddick 2020). Mittal and Singh (2020:2) found similar factors but added that lack of sexual rights among women in making sexual choices can lead to sexual violence by men. Generally, some cases go unreported as more men in telecommutable jobs, which make women to fear calling call-centres for assistance (John et al. 2020:66). Police reluctance to apprehend perpetrators and difficulties in getting restraining orders with the fear of contracting the coronavirus disease 2019 (COVID-19) consequently led to unrecognised, uncounted and unattended GBV cases, leaving the vulnerable groups at risks (John et al. 2020:66).

Globally, the impact of GBV was further aggravated by the outbreak of COVID-19, where the number of reports of violence at call centres has increased from 25% to 50% (Gearin & Knight 2020:1; Ghoshal 2020:1; Graham-Harrison et al. 2020:1; Lundin et al. 2020:1). This led to more deepening of social, economic and gender inequalities among women and children, including sexual exploitation and violence because of breaking of laws during lockdowns (Ghoshal 2020:1). Omotayo and Awoyemi (2021:1) found that many families were strained financially and with increased frustrations, tensions and uncertainty among spouses and partners, particularly in patriarchal cultures. Other serious threats of GBV on health include genital trauma, unwanted pregnancies, shame (Ei & Chuemchit 2020:148), chronic pain, sexual transmitted infections (STIs), depression and other form of cognitive health challenges (Jackson et al. 2002:1).

As per the recommendation of the UN Women and United Nations Population Fund (UNFPA) all affiliated governments had to heed the call to implement the developed guidelines to protect the most vulnerable population (John et al. 2020:67; Lundin et al. 2020:2). In response, several countries such as Chile, Italy, Spain, Argentina, China and the United Kingdom (UK) implemented campaigns targeting sensitisation of the community, and sharing toll-free numbers to the call centres was sanctioned (Lundin et al. 2020:2). Equally, nurses have a critical role in the shaping the behaviour of the community in which they live; however, research found nurses lacking readiness for change affect the quality of care being provided (Kachian et al. 2018:205). Thus, nurses need should be able to respond appropriately by recognising and referring victims for appropriate support (Ali 2018; Poreddi et al. 2020). Nurses should be knowledgeable not only to be able to pose legitimate questions in probing for violence encounters but also in breaking of silence and linking victims where to receive support services (Bradbury-Jones & Isham 2020:1; Liljeroos 2019:29).

In response to GBV in Namibia, various legislations such as Chapter 3 of the *Namibian constitution, Combating of Rape Act* No. 8 of 2000 and *Combating of domestic violence Act* No. 4 of 2003, *Child protection Act* No. 3 of 2015 and *Married persons’ equality Act* No. 1 of 1996 are used to control the surge increase of GBV cases (Haipinge 2021:10; The Republic of Namibia, Ministry of Health and Social Services [MoHSS] 2021:7).

Deliberate efforts were made in Namibia, which included addressing gender inequality through public response interventions such as strengthening partnership with communities, expanding community campaigns on the awareness and reporting incidents of abuse (Dhar 2019:1; Mittal & Singh 2020:3). Although the teaching of GBV has been integrated as part of sexual education for the undergraduate nursing program at the university level, Gender-based violence education is catered for in some nursing modules being Contemporary Social Issues (CSI), Community Health Science (NCH) and General Nursing Science (NGN). Equally, nursing students have a significant role to play in the prevention and management of GBV and are expected to be ready and fully embrace the needed change in behaviour to deal with GBV issues in their practice (Maquibar et al. 2018:1). However, the impact of GBV education in the nursing curriculum in Namibia has not been established despite some studies citing students’ lack of knowledge on the basic competencies in managing GVB (Ei & Chuemchit 2020:149; MoHSS 2021:4). It is, therefore, essential to measure nursing students’ response to issues of GBV within the legal framework on GBV in Namibia. This study assessed undergraduate nursing students’ perceptions and determined the relationship between the study variables to readiness to combat GBV during the COVID-19 pandemic in Namibia.

## Conceptual model

**FIGURE 1:**
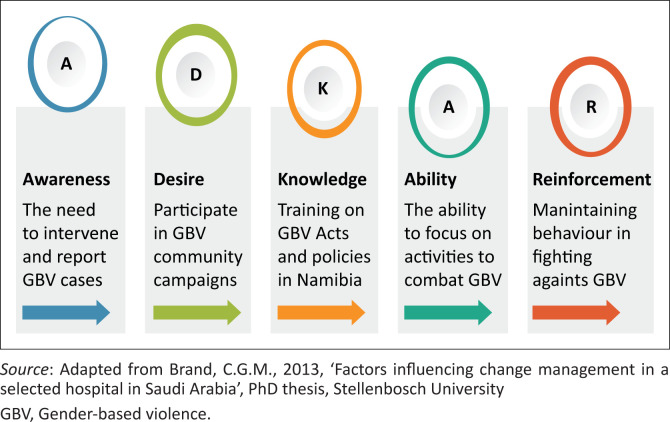
ADKAR model of change behaviour.

There is a dearth of evidence in the literature on the existing tools that measure readiness to fight GBV. Thus, this study measured readiness based on (1) the awareness, (2) desire, (3) knowledge, (4) ability and (5) reinforcement (ADKAR) model, which has been found relevant in determining nursing students’ readiness to fight GBV (Kachian et al. 2018:205). This model, which has been in existence for decades, has been used in hospitals to measure individual behavioural readiness (Brand 2013:1). The existing tools using ADKAR model used ‘yes and no’ questions in measuring behaviour changes (Kachian et al. 2018:205), where yes was used interchangeably as being ready and vice versa. The current study examined the five domains of the ADKAR model to determine the strengths and weaknesses of the undergraduate nursing students in fighting GBV. The *Awareness* assessed the need for change in behaviour, such as the need to intervene and report GBV cases, while *Desire* was viewed as nurses’ interest and support to fight GBV, namely by assessing whether or not respondents participated in community campaigns. *Knowledge* assessed whether or not respondents received training on various Acts and policies in Namibia, whereas *Ability* and *Reinforcement* assessed respondents’ potential to make changes and maintain them (Figure 1).

## Research objectives

The objectives of this study were to:

- assess undergraduate nursing students’ perceptions of their readiness to combat GBV during the COVID-19 pandemic in Namibia
- determine the relationship of the study variables to readiness to combat GBV during the COVID-19 pandemic in Namibia.

## Methods

### Research design and study setting

A cross-sectional research survey was employed to investigate the readiness to combat GBV among undergraduate nursing students. This design has been used in many studies on GBV among health professionals (Ali et al. 2021:1; Beccaria et al. 2013:909). The study was conducted at a satellite university campus located on the eastern part of Namibia, where several GBV cases had been reported. The campus has about 5000 full-time students in the fields of Education, Economics and Nursing Science. The School of Nursing is relatively small school with a population of about 400 undergraduate nursing students. Third and fourth-year nursing students were preferred because of their seniority and closeness to course completion and having completed modules of CSI, NCH and NGN.

### Population and sampling strategy

The target population was 105 third and fourth-year nursing students, recruited from a population of 390 nursing students doing an undergraduate 4-year degree programme at a university satellite campus in Namibia. As described by Sharma (2017:750), the required respondents were recruited using simple random sampling technique as all students stood a chance to be selected in the study. Sample size was determined using Solvin’s formula (Tomas et al. 2022), N/(1 +NXa2), where *N* = total population of undergraduate nursing students in the school of nursing at the university satellite campus in Namibia, using a confidence interval of 95%. About 130 online invitations were sent via students’ university emails. The eligibility criteria were third and fourth year who passed CSI, NCH and NGN modules at first and second year levels where various aspects of GBV has been covered. These students were preferred as they would soon graduate from the programme. The study excluded all senior students who failed both or either CSI, NCH and NGN at first and second year levels.

### Data collection and instrumentation

Data were collected using an online questionnaire developed from existing tools on readiness (Glass et al. 2018:1; Perrin et al. 2019:1). Only aspects that were found to be appropriate to the Namibian setting were selected from the existing tool for this study. These included some parts of the questions and/or question stems. The data collection instrument was developed in English as it was the medium of instruction at the university. The researchers provided a link with vital information on the study objectives and how to proceed with the survey. The use of an online survey was suitable to avoid physical interactions during the COVID-19 pandemic. The scale consisted of two sections; section A included four closed-ended questions on demographic data, whereas section B had 13 yes or no items measuring the five sub-domains of readiness: (1) two items on awareness of GBV behaviour change, (2) four items on desire to fight GBV, (3) three items on knowledge of GBV, (4) single item on ability to implement behaviour change on readiness and (5) three items on reinforcement of GBV-free behaviours. To assess the clarity and consistency of questions and to identify any ambiguity, the instrument was evaluated by a statistician and was piloted on five nursing students who did not form part of the main study. Statistician and pilot students validated the tool and necessitated changes of grammar, clarification and the flow of the questions. In this study, reliability of the tool was calculated with a Cronbach’s alpha coefficient of α = 0.81.

Items on readiness were assigned 2 points for the yes responses and 1 for the no responses. The responses were aggregated to the minimum of 13 (50%) and maximum of 26 (100%), with the highest score indicating a better readiness to fight GBV. Scores were categorised as not ready (0% – 74%) and ready (75% – 100%). Data were collected between 21 and 25 September 2020 with two separate reminders sent via email.

### Data analysis

Data were captured using Google Forms and were analysed using Statistical Package for the Social Sciences, version 27.0 (SPSSv27). Descriptive statistics and inferential statistics were used to analyse data. Logistic regression was performed to determine the relationship between the study variables and students’ readiness to combat GBV at 95% level of significance (*p* < 0.05).

### Ethical considerations

Ethical approval was obtained in writing from the School of Nursing Research Ethics Committee (reference number: SoNEC 65/2020). The consent page was attached as a front page of the questionnaire, where the respondents had to click an ‘agree’ button on the link, after reading the objectives and additional details regarding the study. The consent page also provided information stating that participation was voluntary and that they could withdraw at any time, without any punitive measures were shared on the link. Confidentiality and anonymity were maintained by allowing respondents to complete the questionnaire in the comfort of their homes and excluding respondents’ personal details from the questionnaires.

## Results

Of the 130 invitations sent, only 105 completed questionnaires were returned, giving a completion rate of 80.8%. A low response rate was attributed to that some senior students were repeating NCH II and NGN II, while some did not respond to the reminders sent to them. With the mean age was 25 ± 0.71, the majority of the respondents, 71.4% (*n* = 75), fell into the 18–25 age group, 19% (*n* = 20) into the 26–33 age group, while 7.6% (*n* = 8) fell into the 34–41 age group and 1.9% (*n* = 2) were aged 42 years and above. In terms of gender, the majority of the respondents, 54.3% (*n* = 57), were female compared with 45.7% (*n* = 48) male. More than half, 59% (*n* = 62), of the respondents were in their fourth year of study, while third years comprised just 41% (*n* = 43). In terms of marital status, majority 92.4% (*n* = 97) were unmarried, while only a hand full number 7.6% (*n* = 8) were married. More than two-third, 68.2% (*n* = 73) of the respondent in this study had experienced GBV in the past, while 29.9% (*n* = 32) had not experienced GBV in the past (Table 1).

**TABLE 1:** Demographic data of the respondents (*n* = 105).

Variables	Frequencies (*n*)	%	Mean age
**Age (years)**			25 ± 0.71
18–25	75	71.4	-
26–33	20	19.0	-
34–41	8	7.6	-
˃ 42	2	1.9	-
**Gender**			
Male	48	45.7	-
Female	57	54.3	-
Educational level			-
3rd year	43	59.0	-
4th year	62	41.0	-
**Previous experiences of GBV**			
Yes	73	68.2	-
No	32	29.9	-
**Marital status**			
Married	8	7.6	-
Unmarried	97	92.4	-

GBV, Gender-based violence.

### Nursing students’ readiness to combat gender-based violence

The mean readiness was 1.65 ± 0.19, with 73.3% (*n* = 77) found ready while 26.7% (*n* = 28) were not ready to combat GVB. Readiness was commonly in the subdomain of reinforcement of GBV-free behaviours 89.5% (*n* = 94; 1.83 ± 0.32). Awareness of GBV behaviour change had a readiness of 84.8% (*n* = 89; 1.5 ± 0.32); respondents knowledge of GBV was 81.9% (*n* = 86; 1.77 ± 0.26) with regard to perceiving themselves having adequate knowledge and skills on GBV 80% (*n* = 84) and being well trained to deal with GBV 86.7% (*n* = 91) while desire to fight GBV had a readiness of 76.2% (*n* = 80; 1.55 ± 0.26). Respondents were not ready in the subdomain of the ability to implement behaviour change 54.3% (*n* = 57; 1.54 ± 0.50) (Table 2).

**TABLE 2:** Nursing students’ readiness to combat gender-based violence (*n* = 105).

Variables	Frequencies (*n*)	%	Mean readiness score
**Overall readiness level to combat GBV**			1.65 (0.19)
Ready	77	73.3	-
Not ready	28	26.7	-
**Total**	**105**	**100**	**-**
**Awareness of GBV behaviour change**			1.5 (0.32)
Ready	89	84.8	-
Not ready	16	15.2	-
**Total**	**105**	**100**	**-**
**Desire to fight GBV**			1.55 (0.26)
Ready	80	76.2	-
Not ready	25	23.8	-
**Total**	**105**	**100**	**-**
**Knowledge of GBV**			1.77 (0.26)
Ready	86	81.9	-
Not ready	19	18.1	-
**Total**	**105**	**100**	**-**
**Ability to implement behaviour change**			1.54 (0.50)
Ready	57	54.3	-
Not ready	48	45.7	-
**Total**	**105**	**100**	**-**
**Reinforcement of GBV-free behaviours**			1.83 (0.32)
Ready	94	89.5	-
Not ready	11	10.5	-
**Total**	**105**	**100**	**-**

GBV, Gender-based violence.

### Relationship between respondents’ demographic data and readiness to combat GBV

Table 3 shows the relationship between the study variables (age, gender, marital status, education level age and previous experiences of GBV) and students’ readiness to combat GBV. Predictors of readiness were gender and age with a statistical significance in explaining the total variance of readiness among the respondents in this study (*R*^2^ = 0.40; *R*^2^ = 0.37; *p* ≤ 0.05). Although higher readiness was recorded among married respondents 80% (*n* = 8), who were in fourth years 77.4% (*n* = 48) and those who had no previous experience of GBV 87% (*n* = 67), no statistical significance was found between marital status, education level and previous experiences of GBV (*p* ≥ 0.05).

**TABLE 3:** Predictors of readiness to combat GBV (*n* = 105).

Variable	Readiness to combat GBV				Total		Adjusted *R*^2^	* *p*
	Ready		Not ready		*n*	%		
	*n*	%	*n*	%				
**Age (in years)**								
18–25	48	64	27	36	75	100	0.36	0.00
26–33	9	45	11	55	20	100	-	-
34–41	8	100	0	–	8	100	-	-
˃ 42	2	100	0	–	2	100	-	-
**Total**	**67**	**63.8**	**38**	**36.2**	**105**	**100**	**-**	**-**
**Gender**								
Male	38	79.2	10	20.8	48	100	0.40	0.02
Female	39	68.4	18	31.6	57	100	-	-
**Total**	**77**	**73.3**	**28**	**26.7**	**105**	**100**	**-**	**-**
**Marital status**								
Married	8	80	2	20	10	100	0.00	0.31
Unmarried	63	66.3	32	33.7	95	100	-	-
**Total**	**71**	**67.6**	**34**	**32.4**	**105**	**100**	**-**	**-**
**Education level**								
Third years	26	60.5	17	39.5	43	100	0.00	0.46
Fourth years	48	77.4	14	22.6	62	100	-	-
**Total**	**74**	**70.5**	**31**	**29.5**	**105**	**100**	**-**	**-**
**Previous experiences of GBV**								
Yes	6	21.4	22	78.6	28	100	0.01	0.20
No	67	87	10	13	77	100	-	-
**Total**	**73**	**69.5**	**32**	**30.5**	**105**	**100**	**-**	**-**

GBV, Gender-based violence.

* , *p* < 0.05 = statistically significant.

## Discussion

In assessing undergraduate nursing students’ perception on their readiness to combat GBV during the COVID-19 pandemic in Namibia, the study found a mean average readiness of 1.65 ± 0.19. The majority of the respondents (73.3%) were found to be ready, whereas 26.7% were not ready to combat GVB. The results concur with a study by Natan et al. (2016:1), which found high intentions and preparedness for GBV cases among nursing students. However, our study results had high readiness comparing to a previous study by Ali et al. (2021:1), which found nursing students less knowledgeable and less preparedness to deal with GBV victims.

Readiness was high in the area of being knowledgeable (81.9%). Being knowledgeable on GBV remains a critical strategy of nursing education to enable nurses to become more responsive in their approach in dealing with GBV victims (Kamimura et al. 2015:1453). The results of this study show that the majority (81.9%) of the respondents perceived themselves as knowledgeable about GBV. Common areas of readiness on knowledge were found in possessing adequate knowledge and skills on the Namibian Acts and other important legislations used to combat GBV (80%), with 86.7% of the respondents considering themselves well trained to deal with GBV. Our study results were consistent to Kalra et al. (2021:1) who found that education and training have improved nurses’ attitudes and self-perceived readiness towards GBV victims with a standardised mean difference (SMD) of 0.71, 95 CI 0.39 to 1.03. However, our study results contradicted several studies on GBV found a contradicting results, citing low knowledge owed to neglected education and training on GBV in nursing education despite the importance of education in addressing GBV as a top public health matter (McLindon et al. 2021:1). Ahmad et al. (2017:1) maintain that nurses need appropriate knowledge and skills, while UNFPA (2019:1) stresses that a lack of knowledge on the part of nurses may hinder the successful implementation of a GBV-free society. Our study results are also contradicting the Ministry of Health and Social Services’ (MoHSS) report claiming that there is a lack of knowledge about gender mainstreaming among key stakeholders, in nursing (MoHSS 2021:4). These differences were explained by the lack of training on GBV on existing Acts and policies in other settings (Beccaria et al. 2013:1; Wyatt et al. 2019:1). Some of the studies were used in this studies had other categories of healthcare team, other than the nursing students. Vanner et al. (2022:2) singled out that university need to move away from broader curriculum with a focus on gender inequality, to issues of gender and sexual diversity, if they are to produce nurses with evidence-based competencies in dealing with GBV. Gaps in knowledge also mean that nurses focus more on the physical health of the patient.

The study further observed a high readiness on awareness to change behaviour (84.8%) as well as the desire to fight GBV (76.2%). Maquibar et al. (2018:1) postulate that nurses’ awareness on the need to change their approach towards GBV victims is critical on the social sensitisation and confidence to respond to GBV. In agreement with Kachian et al. (2018:205) study, we found that the desire to make behaviour changes is key to the fight againist GBV. Awareness cannot be divorced from training and other variables such as gender and age. Several studies also found a strong correlation between age and awareness of GBV (Berbegal-Bolsas et al. 2020:149; Redding et al. 2017:1). The difference in findings could be associated to different populations of male and female nursing students and insufficient training provided in other settings (Berbegal-Bolsas et al. 2020:150).

Predictors of readiness were gender and age with a statistical significance in explaining the total variance of readiness among the undergraduate nursing students in this study (*R*^2^ = 0.40; *R*^2^ = 0.37; *p ≤* 0.05). In this study, male were more significantly ready (79.2%; *p* = 0.02) than their female counterpart (68.4%; *p* = 0.02). In alignment with our study findings, Berbegal-Bolsas et al. (2020:149) found that readiness on GBV is associated with feminism (standard deviation: 6.95); however, their findings differed with ours on that female were found more statistically significantly ready than men (*p* = 0.00).

Although reinforcing GBV-free behaviours was high (89.5%) in our study, it cannot be said about in respondents’ ability to implement behaviour change to combat GBV (54.3%). In agreement with our study Rituerto-González et al. (2020:1) states that the fight against GBV is multifaceted and requires a multidisciplinary approaches making it difficult to implement the behaviour changes. Flood (2015:2) suggests that efforts to end GBV should not only condemn overtly condoning behaviours but focus on involving men and their attitudes on gender and sexuality, which is a difficult task for nurses. Crooks et al. (2019:30) maintain that early training on GBV is not always available, and it is key in changing the attitudes and norms that can reduce GBV. Hegarty et al. (2019:302) found that some culture allows for most women to accept being beaten by their partner or regarding beating of a wife or girlfriend an acceptable practice in most communities. In a study on nurses’ perceptions about readiness to manage IPV, Briones-Vozmediano et al. (2021:1457) found that some nurses were not ready to respond to GBV victims because of workload or lack of time.

## Strengths and limitations

The ADKAR model was helpful to identify both areas of strength and weakness of this study. Accordingly, as Lundin et al. (2020:2) subscribe, nursing students are part of social and health services can assist in improving the surveillance in the fight against GBV. The results of this study will support in strengthening the health system response and global efforts in combating GBV. Clearly, the results of this study can be used as data base in Namibia to evaluate the effectiveness of existing GBV education integrated in the nursing curriculum at university level. Assessing the readiness of nursing students is key not only in measuring their efforts but their readiness to fight against GBV in their career as nurses. One of the major limitations of this study was that the data were self-reported and thus should be interpreted with caution. Owed to a relative small sample size of this study, the generalisability of the results is limited one setting. Including other sister campuses proven difficult as data were collected during COVID-19 lockdown. It would be possible for students from campuses who had interest in GBV could have participated in the survey.

## Conclusion

This study assessed undergraduate nursing students’ perceptions of their readiness and determine the relationship of the study variables to readiness to combat GBV during the COVID-19 pandemic in Namibia. The results of this study highlight that age and gender were significant predictors for readiness among the undergraduate students in Namibia. The results highlight the importance of GBV education in a nursing curriculum. However, respondents were not ready to implement behaviour change, in the fight against GBV. In support of the study results, Oliveira et al. (2020:1) stated that upholding nursing values is vital for change in behaviours, adapting to social changes and being devoted to serve as a nurse. It is recommended for future studies to increase the scope of the study and a qualitative study would provide deep insight and understanding of the factors influencing readiness to combat GBV.

## References

[CIT0001] Ahmad, I., Ali, P.A., Rehman, S., Talpur, A. & Dhingra, K., 2017, ‘Intimate partner violence screening in emergency department: A rapid review of the literature’, *Journal of Clinical Nursing* 26(21–22), 3271–3285. 10.1111/jocn.13706 28029719

[CIT0002] Ali, P., 2018, ‘Gender-based violence and the role of healthcare professionals’, *Nursing Open* 5(1), 4. 10.1002/nop2.120 29344388 PMC5762759

[CIT0003] Ali, P., McGarry, J., Younas, A., Inayat, S. & Watson, R., 2021, ‘Nurses’, midwives’ and students’ knowledge, attitudes and practices related to domestic violence: A cross-sectional survey’, *Journal of Nursing Management*. 10.1111/jonm.13503 34734662

[CIT0004] Amakali, M., 2021, ‘400 GBV cases withdrawn in 2020’, *New Era*, viewed 10 June 2022, from https://neweralive.na/posts/400-gbv-cases-withdrawn-in-2020.

[CIT0005] Beccaria, G., Beccaria, L., Dawson, R., Gorman, D., Harris, J.A. & Hossain, D., 2013, ‘Nursing student’s perceptions and understanding of intimate partner violence’, *Nurse Education Today* 33(8), 907–911. 10.1016/j.nedt.2012.08.004 23021564

[CIT0006] Berbegal-Bolsas, M., Gasch-Gallén, Á., Oliván-Blázquez, B., Calavera, M.A.S., García-Arcega, P. & Magallón-Botaya, R., 2020, ‘Variables associated with a higher awareness of gender-based violence by students of the health sciences and social work’, *Gaceta Sanitaria* 36(2), 146–151. 10.1016/j.gaceta.2020.09.005 33131905

[CIT0007] Beyene, A.S., Chojenta, C., Roba, H.S., Melka, A.S. & Loxton, D., 2019, ‘Gender-based violence among female youths in educational institutions of sub-Saharan Africa: A systematic review and meta-analysis’, *Systematic Reviews* 8(1), 1–14. 10.1186/s13643-019-0969-9 30803436 PMC6388495

[CIT0008] Bradbury-Jones, C. & Isham, L., 2020, ‘The pandemic paradox: The consequences of COVID-19 on domestic violence’, *Journal of Clinical Nursing* 29(13–14), 2047–2049. 10.1111/jocn.15296 32281158 PMC7262164

[CIT0009] Brand, C.G.M., 2013, ‘Factors influencing change management in a selected hospital in Saudi Arabia’, PhD thesis, Stellenbosch University.

[CIT0010] Briddick, C., 2020, ‘Combatting or enabling domestic violence? Evaluating the residence rights of migrant victims of domestic violence in Europe’, *International & Comparative Law Quarterly* 69(4), 1013–1034. 10.1017/S0020589320000317

[CIT0011] Briones-Vozmediano, E., Otero-García, L., Gea-Sánchez, M., De Fuentes, S., García-Quinto, M., Vives-Cases, C. et al., 2021, ‘A qualitative content analysis of nurses’ perceptions about readiness to manage intimate partner violence’, *Journal of Advanced Nursing* 78(5), 1448–1460. 10.1111/jan.15119 34854496

[CIT0012] Buttell, F. & Ferreira, R.J., 2020, ‘The hidden disaster of COVID-19: Intimate partner violence’, *Psychological Trauma: Theory, Research, Practice, and Policy* 12(S1), S197. 10.1037/tra0000646 32567875

[CIT0013] Chime, O.H., Nduagubam, O.C. & Orji, C.J., 2022, ‘Prevalence and patterns of gender-based violence in Enugu, Nigeria: A cross-sectional study’, *The Pan African Medical Journal* 41, 198. 10.11604/pamj.2022.41.198.29454 35685097 PMC9146663

[CIT0014] Crooks, C.V., Jaffe, P., Dunlop, C., Kerry, A. & Exner-Cortens, D., 2019, ‘Preventing gender-based violence among adolescents and young adults: Lessons from 25 years of program development and evaluation’, *Violence Against Women* 25(1), 29–55. 10.1177/1077801218815778 30803428

[CIT0015] Dhar, S., 2019, ‘Gender and Sustainable Development Goals (SDGs)’, *Indian Journal of Gender Studies* 25(1), 47–78. 10.1177/0971521517738451

[CIT0016] Dlamini, N.J., 2021, ‘Gender-based violence, twin pandemic to COVID-19’, *Critical Sociology* 47(4–5), 583–590. 10.1177/0896920520975465 PMC7723732 38603014

[CIT0017] Ei, A.N. & Chuemchit, M., 2020, ‘How prepared are Myanmar’s health care professionals in their response to gender-based violence?’, *Journal of Health Research* 35(2), 147–159. 10.1108/JHR-08-2019-0188

[CIT0018] Engeli, I. & Mazur, A., 2018, ‘Taking implementation seriously in assessing success: The politics of gender equality policy’, *European Journal of Politics and Gender* 1(1–2), 111–129. 10.1332/251510818X15282097548558

[CIT0019] Ertan, D., El-Hage, W., Thierrée, S., Javelot, H. & Hingray, C., 2020, ‘COVID-19: Urgency for distancing from domestic violence’, *European Journal of Psychotraumatology* 11(1), 1800245. 10.1080/20008198.2020.1800245 33110483 PMC7560728

[CIT0020] Flood, M., 2015, ‘Work with men to end violence against women: A critical stocktake’, *Culture, Health & Sexuality* 17(suppl. 2), 159–176. 10.1080/13691058.2015.1070435 PMC4706022 26414870

[CIT0021] Gearin, M. & Knight, B., 2020, ‘Family violence perpetrators using COVID-19 as a form of abuse we have not experienced before’, *ABC News*, p. 28, viewed 03 August 2022, from https://www.abc.net.au/news/2020-03-29/coronavirus-family-violence-surge-invictoria/12098546.

[CIT0022] Ghoshal, R., 2020, ‘Twin public health emergencies: COVID-19 and domestic violence’, *Indian Journal of Medical Ethics* 5(3), 195–199. 10.20529/IJME.2020.056 32546463

[CIT0023] Glass, N., Perrin, N., Clough, A., Desgroppes, A., Kaburu, F.N., Melton, J. et al., 2018, ‘Evaluating the communities care program: Best practice for rigorous research to evaluate gender based violence prevention and response programs in humanitarian settings’, *Conflict and Health* 12(1), 1–10. 10.1186/s13031-018-0138-0 29422946 PMC5791214

[CIT0024] Graham-Harrison, E., Giuffrida, A., Smith, H. & Ford, L., 2020, ‘Lockdowns around the world bring rise in domestic violence’, *The Guardian*, viewed 10 June 2022, from https://www.theguardian.com/society/2020/mar/28/lockdowns-world-rise-domestic-violence

[CIT0025] Haipinge, M., 2021, ‘A critique on gender-based violence legislation in Namibia and Zambia’, Doctoral dissertation, Cavendish University.

[CIT0026] Hegarty, K., Tarzia, L., Valpied, J., Murray, E., Humphreys, C., Taft, A. et al., 2019, ‘An online healthy relationship tool and safety decision aid for women experiencing intimate partner violence (I-DECIDE): A randomised controlled trial’, *The Lancet Public Health* 4(6), e301–e310. 10.1016/S2468-2667(19)30079-9 31155223

[CIT0027] Jackson, H., Philp, E., Nuttall, R.L. & Diller, L., 2002, ‘Traumatic brain injury: A hidden consequence for battered women’, *Professional Psychology: Research and Practice* 33(1), 39–45. 10.1037/0735-7028.33.1.39

[CIT0028] John, N., Casey, S.E., Carino, G. & McGovern, T., 2020, ‘Lessons never learned: Crisis and gender-based violence,’ *Developing World Bioethics* 20(2), 65–68. 10.1111/dewb.12261 32267607 PMC7262171

[CIT0029] Kachian, A., Elyasi, S. & Haghani, H., 2018, ‘ADKAR model and nurses’ readiness for change’, *Journal of Client-Centered Nursing Care* 4(4), 203–212. 10.32598/jccnc.4.4.203

[CIT0030] Kagi, J., 2020, ‘Crime rate in WA plunges amid coronavirus social distancing lockdown measures’, *ABC News Australia*, viewed 09 June 2022, from https://www.abc.net.au/news/2020-04-08/coronavirus-shutdown-sees-crime-ratedrop-in-wa/12132410.

[CIT0031] Kalra, N., Hooker, L., Reisenhofer, S., Di Tanna, G.L. & Garcia-Moreno, C., 2021, ‘Training healthcare providers to respond to intimate partner violence against women’, *Cochrane Database of Systematic Reviews* 2, CD012423. 10.1002/14651858.CD012423.pub2 PMC8166264 34057734

[CIT0032] Kamimura, A., Al-Obaydi, S., Nguyen, H., Trinh, H.N., Mo, W., Doan, P. et al., 2015, ‘Intimate partner violence education for medical students in the USA, Vietnam and China’, *Public Health* 129(11), 1452–1458. 10.1016/j.puhe.2015.04.022 26047798

[CIT0033] Liljeroos, T., 2019, ‘Caring for migrant women affected by sexual and gender-based violence: Experiences of healthcare providers in Europe and North America: A meta-synthesis’, Master’s thesis, Uppsala University.

[CIT0034] Lundin, R., Armocida, B., Sdao, P., Pisanu, S., Mariani, I., Veltri, A. et al., 2020, ‘Gender-based violence during the COVID-19 pandemic response in Italy’, *Journal of Global Health* 10(2), 20359. 10.7189/jogh.10.020359 PMC7568007 33110555

[CIT0035] Mak, I.W.C., Chu, C.M., Pan, P.C., Yiu, M.G.C. & Chan, V.L., 2009, ‘Long-term psychiatric morbidities among SARS survivors’, *General Hospital Psychiatry* 31(4), 318–326. 19555791 10.1016/j.genhosppsych.2009.03.001 PMC7112501

[CIT0036] Maquibar, A., Hurtig, A.K., Vives-Cases, C., Estalella, I. & Goicolea, I., 2018, ‘Nursing students’ discourses on gender-based violence and their training for a comprehensive healthcare response: A qualitative study’, *Nurse Education Today* 68, 208–212. 10.1016/j.nedt.2018.06.011 29966882

[CIT0037] McLindon, E., Fiolet, R. & Hegarty, K., 2021, ‘Is gender-based violence a neglected area of education and training? An analysis of current developments and future directions’, In: Bradbury-Jones, C., Isham, L. (eds.), *Understanding gender-based violence*, pp. 15–30, Springer, Cham. 10.1007/978-3-030-65006-3_2

[CIT0038] Mittal, S. & Singh, T., 2020, ‘Gender-based violence during COVID-19 pandemic: A mini-review’, *Frontiers in Global Women’s Health* 1, 4. 10.3389/fgwh.2020.00004 PMC8594031 34816149

[CIT0039] Montesanti, S.R., 2015, ‘The role of structural and interpersonal violence in the lives of women: A conceptual shift in prevention of gender-based violence’, *BMC Women’s Health* 15(1), 1–3. 10.1186/s12905-015-0247-5 26503594 PMC4623903

[CIT0040] Mukaiwa, M., 2019, ‘The effect of the pandemic on Namibian women’, *Sister Namibia*, viewed 22 June 2022, from https://sisternamibia.org/2021/06/02/the-effect-of-the-pandemic-on-namibian-women/.

[CIT0041] Muluneh, M.D., Stulz, V., Francis, L. & Agho, K., 2020, ‘Gender based violence against women in sub-Saharan Africa: A systematic review and meta-analysis of cross-sectional studies’, *International Journal of Environmental Research and Public Health* 17(3), 903. 10.3390/ijerph17030903 32024080 PMC7037605

[CIT0042] Natan, M.B., Khater, M., Ighbariyea, R. & Herbet, H., 2016, ‘Readiness of nursing students to screen women for domestic violence’, *Nurse Education Today* 44, 98–102. 10.1016/j.nedt.2016.05.006 27429336

[CIT0043] Noble, E., Ward, L., French, S. & Falb, K., 2019, ‘State of the evidence: A systematic review of approaches to reduce gender-based violence and support the empowerment of adolescent girls in humanitarian settings’, *Trauma, Violence, & Abuse* 20(3), 428–434. 10.1177/1524838017699601 29334024

[CIT0044] Oliveira, K.K.D.D., Freitas, R.J.M.D., Araújo, J.L.D. & Gomes, J.G.N., 2020, ‘Nursing now and the role of nursing in the context of pandemic and current work’, *Revista Gaúcha de Enfermagem* 42, e20200120. 10.1590/1983-1447.2021.20200120 33084790

[CIT0045] Omotayo, O. & Awoyemi, B.O., 2021, ‘Covid-19 pandemic, violence and social inequality in Nigeria’, *International Journal of Social Science and Human Research* 4(9), 2600–2603. 10.47191/ijsshr/v4-i9-46

[CIT0046] Paganetti, L., 2022, ‘Europe can succeed where America failed: A comparative approach to gender-based violence’, *Columbia Journal of Gender and Law*. (2019), 1–39. 10.2139/ssrn.4069636

[CIT0047] Perrin, N., Marsh, M., Clough, A., Desgroppes, A., Yope Phanuel, C., Abdi, A. et al., 2019, ‘Social norms and beliefs about gender based violence scale: A measure for use with gender based violence prevention programs in low-resource and humanitarian settings’, *Conflict and Health* 13(1), 1–12. 10.1186/s13031-019-0189-x 30899324 PMC6408811

[CIT0048] Poreddi, V., Gandhi, S., Palaniappan, M. & BadaMath, S., 2020, ‘Violence against women with mental illness and routine screening: Nurses’ knowledge, confidence, barriers and learning needs’, *Archives of Psychiatric Nursing* 34(5), 398–404. 10.1016/j.apnu.2020.07.015 33032765

[CIT0049] Redding, E.M., Ruiz-Cantero, M.T., Fernández-Sáez, J. & Guijarro-Garvi, M., 2017, ‘Gender inequality and violence against women in Spain, 2006–2014: Towards a civilized society’, *Gaceta Sanitaria* 31(2), 82–88. 10.1016/j.gaceta.2016.07.025 27793548 PMC5858551

[CIT0050] Rituerto-González, E., Miranda, J.A., Canabal, M.F., Lanza-Gutiérrez, J.M., Peláez-Moreno, C. & López-Ongil, C., 2020, ‘A hybrid data fusion architecture for BINDI: A wearable solution to combat gender-based violence’, In: Dziech, A., Mees, W., Czyżewski, A. (eds.), *International Conference on Multimedia Communications, Services and Security*, pp. 223–237, Springer, Cham. 10.1007/978-3-030-59000-0_17

[CIT0051] Sharma, G., 2017, ‘Pros and cons of different sampling techniques’, *International Journal of Applied Research* 3(7), 749–752.

[CIT0052] The Republic of Namibia, Ministry of Health and Social Services, 2021, *16 days of activism against gender-based violence*, viewed 22 June 2022, from https://mgepesw.gov.na/documents/792320/0/Newsletter+Quarter+3+of+2021-22.pdf/c685657d-7a54-87b8-3e68-793e17acf939.

[CIT0053] Tomas, N., Ndjamba, A.K. & Munangatire, T., 2021, ‘Undergraduate nursing students’ self-reported professional behaviour at the University of Namibia’, *Health SA Gesondheid* 26(0), 1–7. 10.4102/hsag.v26i0.1703 PMC8661282 34917406

[CIT0054] UNFPA, 2019, *Health workers can treat, empower survivors of gender-based violence*, viewed 12 December 2020, from https://www.unfpa.org/news/health-workers-can-treat-empower-survivors-gender-based-violence.

[CIT0055] Union, I.P., 2018, *Sexism, harassment and violence against women in parliaments in Europe*, viewed 09 June 2022, from https://dspace.ceid.org.tr/xmlui/bitstream/handle/1/872/IPU%20siyasette%20cinsiyet%C3%A7ilik%20raporu%20en_2018-issues_brief_web.pdf?sequence=1&isAllowed=y.

[CIT0056] UN Women, 2020, *The shadow pandemic: Violence against women during COVID-19*, viewed 02 April 2022, from https://www.unwomen.org/en/news/in-focus/in-focus-gender-equality-in-covid-19-response/violence-against-women-during-covid-19.

[CIT0057] Vanner, C., Holloway, A. & Almanssori, S., 2022, ‘Teaching and learning with power and privilege: Student and teacher identity in education about gender-based violence’, *Teaching and Teacher Education* 116, 1–11. 10.1016/j.tate.2022.103755

[CIT0058] Wyatt, T., McClelland, M.L. & Spangaro, J., 2019, ‘Readiness of newly licensed associated degree registered nurses to screen for domestic violence’, *Nurse Education in Practice* 35, 75–82. 10.1016/j.nepr.2018.12.010 30716539

